# An ICEEMDAN and SAX-based method for determining English reading comprehension status using functional near-infrared spectroscopy signals

**DOI:** 10.1371/journal.pone.0326359

**Published:** 2025-07-23

**Authors:** Ural Akincioglu, Onder Aydemir, Ahmet Cil, Muhammed Baydere

**Affiliations:** 1 Electronics and Communication Engineering Department of Faculty of Technology, Karadeniz Technical University, Trabzon, Türkiye; 2 Electrical and Electronics Engineering Department, Engineering Faculty, Karadeniz Technical University, Trabzon, Türkiye; 3 Medical Device and Production Application and Research Center, Karadeniz Technical University, Trabzon, Türkiye; 4 Department of Western Languages and Literature, Faculty of Literature, Karadeniz Technical University, Trabzon, Türkiye; Air University, PAKISTAN

## Abstract

Accurate, rapid, and objective reading comprehension assessments, which are critical in both daily and educational lives, can be effectively conducted using brain signals. In this study, we proposed an improved complementary ensemble empirical mode decomposition with adaptive noise (ICEEMDAN) and symbolic aggregate approximation (SAX)-based method for determining the whole text reading comprehension status in English using functional near-infrared spectroscopy (fNIRS) signals. A total of 450 trials were recorded from 15 healthy participants as they read English texts. To facilitate labeling, participants were asked to rate their comprehension of the text using self-assessment scores, followed by answering a multiple-choice question with four options that comprehensively covered the whole text’s content. The proposed method consists of pre-processing, feature extraction, and classification stages. In the pre-processing stage, intrinsic mode functions of the signals were obtained using the ICEEMDAN algorithm. In the feature extraction stage, following the SAX algorithm, statistical features were calculated. The extracted features were classified using the *k*-NN classifier. The proposed method tested three different labeling strategies: first, labeling the trials according to the responses to multiple-choice questions; second, labeling the trials based on self-assessment scores; and third, labeling the trials using a double-validation labeling strategy based on the intersection sets of the first two strategies. For the three strategies, the *k*-NN classifier achieved mean classification accuracies of 74.67%, 66.37%, and 89.02%, respectively. The results indicated that the proposed method could assess whole-text reading comprehension status in English.

## Introduction

Reading comprehension is an active and dynamic cognitive process in which readers process textual information to construct meaningful mental representations and integrate the content with their existing knowledge [1]. This process requires readers to interpret written text, actively engage with it, and make inferences based on prior knowledge, experience, and contextual cues [2]. It extends beyond extracting word-level meanings; readers utilize textual cues and their background knowledge to establish causal relationships and construct a coherent understanding of the text as a whole [3]. Given the complexity of this process, researchers have sought to investigate the cognitive and neural mechanisms underlying reading comprehension using various neuroimaging techniques.

Recent advancements in neuroscience have enabled the exploration of brain activity associated with reading comprehension through functional neuroimaging methods such as functional magnetic resonance imaging (fMRI), electroencephalography (EEG), and magnetoencephalography (MEG). These techniques have provided valuable insights into the neural networks involved in language processing and comprehension. In an EEG study, Yuan *et al*. [4] tried to predict reading comprehension using the EEG method and machine learning. The realized system predicted reading comprehension with a classification accuracy (CA) rate of approximately 60%. In a MEG study, neural activity was measured during the comprehension of simple adjective-noun sentences, and the same linguistic materials were used in both reading and listening conditions. As a result, common neural mechanisms were determined in the left anterior temporal lobe and left angular gyrus during reading and listening [5]. fMRI studies have consistently highlighted increased activity in the left inferior frontal gyrus, left temporo-parietal cortex, and left occipito-temporal region during reading tasks [6]. Furthermore, Yang *et al*. [7] demonstrated that reading meaningful words elicits significant neural activity in semantic processing areas such as the superior temporal gyrus and inferior frontal gyrus.

Despite their valuable contributions, these neuroimaging methods have certain limitations. fMRI provides high spatial resolution but has low temporal resolution, whereas EEG and MEG offer excellent temporal resolution but suffer from limited spatial resolution. Additionally, these techniques are costly and impose constraints on participant movement, which can limit their applicability in naturalistic reading studies. Compared to other neuroimaging techniques, functional near-infrared spectroscopy (fNIRS) has emerged as a promising neuroimaging modality that balances spatial and temporal resolution, offers portability, and demonstrates greater tolerance to motion artifacts, making it particularly well-suited for studying cognitive processes such as reading comprehension [8–12].

fNIRS measures brain activity by detecting hemodynamic changes in the cerebral cortex, specifically tracking variations in oxygenated (OxyHb) and deoxygenated hemoglobin (DeoxyHb) concentrations, which serve as indirect indicators of neuronal activity [13]. Several studies have utilized fNIRS to investigate reading comprehension. For instance, Kahlaoui *et al*. [14] used fNIRS to investigate hemispheric differences during word and pseudoword reading, revealing increased blood oxygenation in both hemispheres when processing pseudowords. Similarly, Safi *et al*. [15] examined changes in brain function while participants were reading meaningful and meaningless words. As a result, the participants assigned more brain function to meaningful words in their native language than meaningless words. Midha *et al*. [16] examined fNIRS data obtained at different levels of reading difficulty. The results support the findings that changes in mental workload for more complex reading tasks are associated with increased neural activity and are detectable in the prefrontal cortex (PFC). Reading comprehension is strongly related to the PFC (besides temporal regions), i.e., Broca’s area located in the left inferior frontal gyrus, and thus there is agreement that the left hemisphere is dominant in reading comprehension [17–19].

Although previous studies have established strong associations between brain activity and reading comprehension, most have focused on regional brain activation during word recognition, sentence processing, or text comprehension. However, few studies have explored machine learning-based classification models to determine reading comprehension status using neuroimaging data. Developing such a classification approach would be valuable for automated cognitive assessment, personalized learning, and adaptive educational technologies. Furthermore, neural potentials for real-time classification remain underexplored. Given the advantages of fNIRS and the increasing role of machine learning in neuroscience, we hypothesize that it is feasible to determine the overall reading comprehension status for a whole English text using fNIRS.

Therefore, in this study, we propose an improved complementary ensemble empirical mode decomposition with adaptive noise (ICEEMDAN) and symbolic aggregate approximation (SAX)-based approach to classify reading comprehension as either *understood* or *not understood* based on fNIRS signals. ICEEMDAN enhances signal decomposition by reducing noise and improving feature extraction, while SAX enables efficient pattern recognition in neurophysiological time-series data. Unlike traditional feature extraction and classification methods, our approach leverages the complementary strengths of ICEEMDAN and SAX, leading to improved signal clarity and pattern detection in complex neurophysiological data. Compared to conventional models, our approach enhances signal robustness and provides a more precise distinction between comprehension states, making it particularly suitable for real-time cognitive assessment. By integrating advanced signal processing and machine learning techniques, this study aims to contribute to the growing body of research on reading comprehension and its neural basis while offering a novel, automated method for assessing comprehension levels using neurophysiological data.

The machine learning model in our approach is designed to effectively capture patterns in fNIRS signals associated with comprehension status. Furthermore, the classification model employs supervised learning techniques to train on labeled fNIRS datasets, enabling it to generalize to new data and predict whether a reader has understood a given text. This capability has significant implications for cognitive neuroscience, as it allows real-time and automated assessment of reading comprehension without reliance on subjective self-reports or behavioral tests. The integration of these techniques can enhance educational tools by providing personalized feedback, identifying reading difficulties early, and adapting instructional content based on individual cognitive processing patterns. Additionally, this approach distinguishes itself by incorporating ICEEMDAN and SAX, which together offer improved feature extraction and pattern recognition compared to traditional models. Ultimately, this study paves the way for future research in neuroeducational applications and machine learning-driven cognitive assessments.

## Materials and methods

### Participants

The fNIRS signals were recorded from 15 healthy participants (9 men and 6 women) with a mean age of 29.20 ± 4.26 years. In the participant selection process, the scores obtained by participants in the Foreign Language Proficiency Exam of Turkey, which is used for academic and professional evaluations, were taken into account to ensure an approximately uniform distribution of English proficiency levels. As a result, the study sample comprised participants with varying levels of English proficiency.

Prior to the experiment, participants completed a demographic questionnaire, providing their personal information and confirming English as their second language. This step ensured that all participants met the study’s inclusion criteria concerning language background and proficiency levels. All participants had normal vision, and none reported a history of neurological or psychiatric disorders.

The participants were required to sign written informed consent documents before the experimental tests. All participants were volunteers and were not offered financial compensation for participation. The Karadeniz Technical University Faculty of Medicine Scientific Research Ethics Committee approved the data collection process. The recruitment period for this study started on September 13, 2022, and ended on December 12, 2022.

### Materials

A total of 30 English reading texts were carefully selected to represent a spectrum of comprehension difficulties. Each text contained between 48 and 77 words and comprised 3 to 10 sentences. To address parameters widely recognized as influencing reading difficulty, we carefully managed lexical complexity, syntactic structure, and semantic clarity. Lexical complexity was controlled by selecting a corpus of texts that featured a balanced range of vocabulary in terms of frequency and difficulty, ensuring that less familiar words were represented alongside common ones. Syntactic structure varied in terms of sentence length, clause complexity, and syntactic embedding, including texts with differing grammatical constructions. Semantic clarity was ensured by pre-testing passages to confirm that the intended meanings were conveyed unambiguously. Consistent with these parameters and to reflect diverse real-world reading contexts, we incorporated both expository and narrative texts, covering topical themes such as technology, social and cultural issues, environmental and economic debates, education and learning, personal narratives, historical contexts, cross-cultural perspectives, animal behavior, and mystery narrative. To ensure that our corpus offered a varied readability experience reflecting different levels of text difficulty, we assessed the readability of each text using the Flesch Reading Ease Score. The scores of the texts ranged from 6.4 to 98.94, resulting in the categorization of 4 texts as ‘very easy,’ 4 as ‘easy,’ 3 as ‘fairly easy,’ 7 as ‘standard,’ 4 as ‘fairly difficult,’ 6 as ‘difficult,’ and 2 as ‘very difficult.’ Each reading text was paired with a multiple-choice question aimed at assessing overall comprehension. The texts and accompanying questions were prepared in close collaboration with the Department of Western Languages and Literature at Karadeniz Technical University, ensuring that the abovementioned criteria were met.

### Experimental procedure

**Fig 1:**
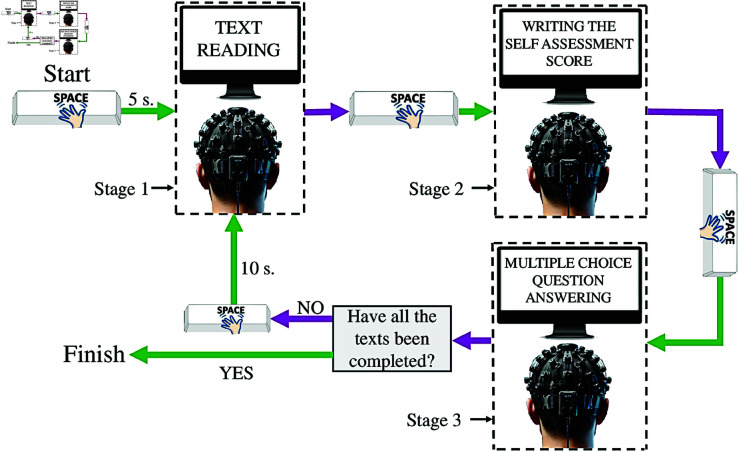
Experimental flowchart.

The participants, seated in a comfortable chair in front of the LED display and wearing the fNIRS device, received information about the experiment by reading the participant information guide. This guide highlighted essential points in addition to information about the experiment flow. One conveyed that they could leave answers blank to avoid random marking, increasing the system’s reliability. Another point assured the participants that nobody would make any weird comments in the event of incorrect answers so that they would not feel pressure and stress and would mark what they understood. Before starting the experiment, an answer sheet on which to write self-assessment scores (SAS) and answers to the multiple-choice questions was given to the participants. The reading experiment consisted of three stages. In Stage 1, the participants read a text; in Stage 2, they wrote the SAS; in Stage 3, they answered the multiple-choice question related to the text on the answer sheet. These three stages were repeated for 30 different texts. The multiple-choice questions were carefully prepared to cover the full content of the text. An experimental flowchart is shown in Fig 1. The experimental presentation was conducted using the Psychtoolbox in Matlab R2022a. The experiment began when participants pressed the space key to indicate they were ready, following which a 5-second countdown was displayed before the first text appeared on the monitor. Participants read the text at their own pace without any time constraints. The only rule was to read each word only once, progressing forward without going back. Upon completing the reading, participants pressed the space key again, prompting the self-assessment stage to appear on the screen. The participants rated their understanding on a scale from 1 to 10 during this stage. If the participants believed they understood the text poorly, they assigned SAS between 1 and 4. For a medium level of understanding, SAS ranged from 5 to 7, while SAS between 8 and 10 were used if they believed they understood the text well. They wrote the scores in the SAS section of the answer sheet. After the self-assessment stage, when the space button was pressed again, the multiple-choice question appeared on the screen. During this stage, the participants read the question and four answer options without restrictions and wrote their answers on the answer key. When the space button was pressed after the multiple-choice question stage, the new text appeared on the screen after a 10-second countdown. These steps were the same for 30 texts. With the help of Psychtoolbox, the starting and ending times of the texts were automatically marked in the fNIRS signal recordings. The accuracy of this marking is crucial for the study. Furthermore, by synchronizing the brain signal recording and the experimental presentation, the experiment was performed automatically in an isolated room with only the participant and the LED display, without any external distractions.

### fNIRS recording

**Fig 2:**
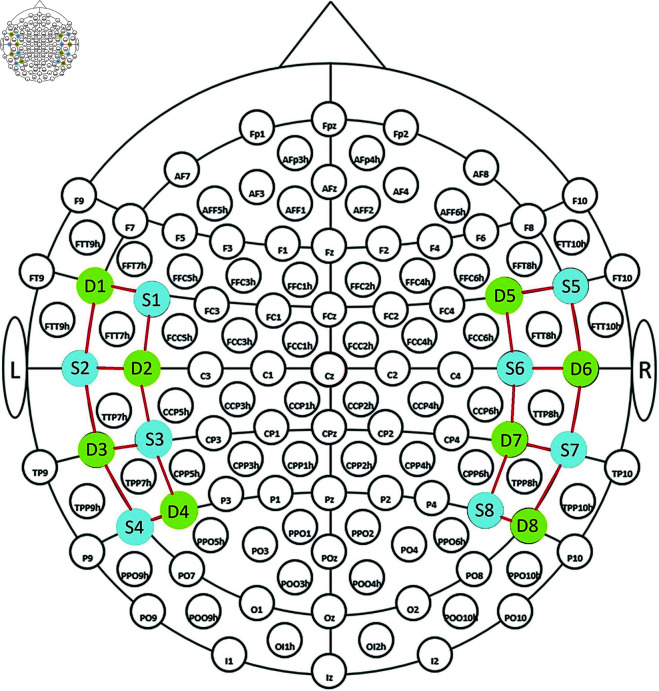
Detectors, sources, and channels.

In the experiment, fNIRS signals were captured using the NIRX NIRSport2 device. Data were recorded from 20 channels, including eight sources and eight detectors from the brain’s temporal lobe at a sampling frequency of 10.1725 Hz. The positions of detectors and sources on the fNIRS cap are shown in Fig 2. In this figure, the detectors are shown in green, the sources in blue, and the channels between the detectors and the sources are shown with red lines. Due to the limited number of detectors and sources, only one brain lobe could be examined. The selection of channel locations was based on anatomical and functional considerations informed by prior neuroimaging studies on reading comprehension tasks [5–7]. Channels covering the left and right temporal cortices were included, given the well-established involvement of the superior temporal gyrus and neighboring areas in language comprehension tasks. This selection was intended to maximize the sensitivity to hemodynamic changes associated with reading comprehension processes.

**Fig 3:**
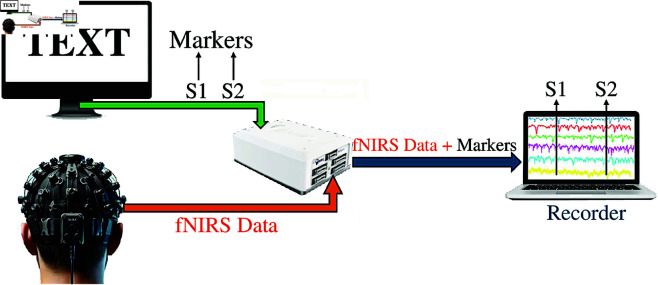
fNIRS signals recording procedure.

The data collection setup consists of a fNIRS device and two computers, one for experimental presentation and one for data recording, as shown in Fig 3. For each text, separately, the starting of the text was marked as S1, and the ending of the text was marked as S2 in the fNIRS signals. The fNIRS signals within the range S1 to S2 were defined as trials and utilized in this study. A total of 450 trials were recorded from 15 participants.

### The proposed method

**Fig 4:**
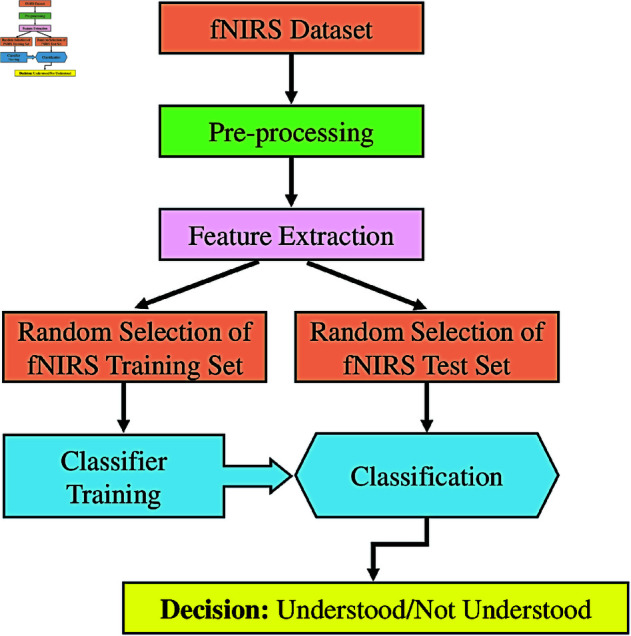
Block diagram of the proposed method.

The proposed method consists of pre-processing, feature extraction, and classification steps, and its block diagram is presented in Fig 4. The experimental presentation is shown on an LED display to the participant in the beginning. Corresponding brain activities are collected simultaneously using an fNIRS cap worn by the participant to monitor their brain response to the reading text, and they are recorded. After the fNIRS data collection and recording, linear interpolation and ICEEMDAN are applied in the pre-processing stage. Statistical features are extracted following SAX in the feature extraction stage. In the final stage, these extracted features are classified using the *k*-nearest neighbor (*k*-NN) classifier. The steps related to the proposed method are detailed below.

#### Signal pre-processing

**Fig 5:**
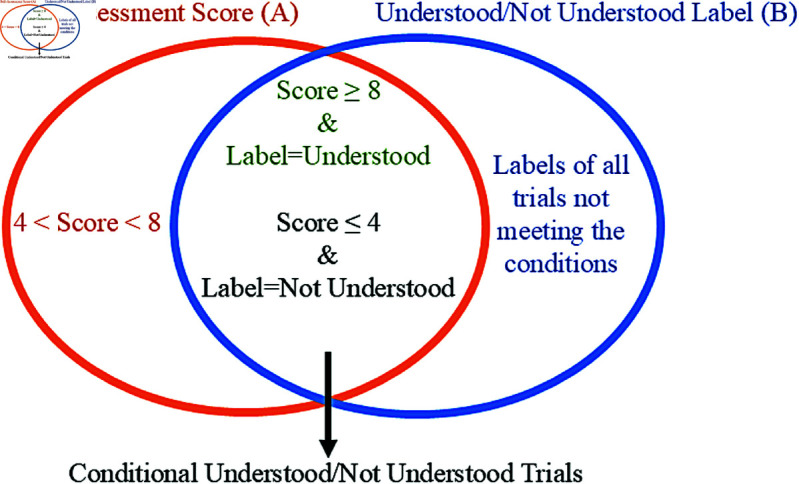
Labeling strategy.

We tested the proposed method separately for three strategies, each established based on the labeling strategy, as illustrated in Fig 5. In the first strategy, represented by Set B in Fig 5, the recorded 450 trials were labeled as either *understood* or *not understood* based on the accuracy of the answers given to multiple-choice questions. In the second strategy, illustrated by Set A in Fig 5, 450 trials were labeled into three classes based on their SAS. The class labels were as follows: Class *1* (*not understood*) for SAS between 1 and 4, class *2* (*a little understood*) for SAS between 5 and 7, and class *3* (*understood*) for SAS between 8 and 10. In the third strategy, we aimed to obtain double-validated labeled trials to enhance the accuracy of labeling as *understood* or *not understood* and to minimize errors caused by random correct or incorrect answers. We selected trials with a SAS of 8 or higher, labeled as *understood*, and trials with a SAS of 4 or lower, labeled as *not understood*. These trials were referred to as conditional *understood/not understood* trials, respectively. The third strategy is expressed as the Set A∩B in Fig 5. Consequently, 274 out of 450 trials met the criteria of the conditional *understood*/*not understood* labeling strategy, resulting in the acquisition of double-validated labeled trials.

**Fig 6:**
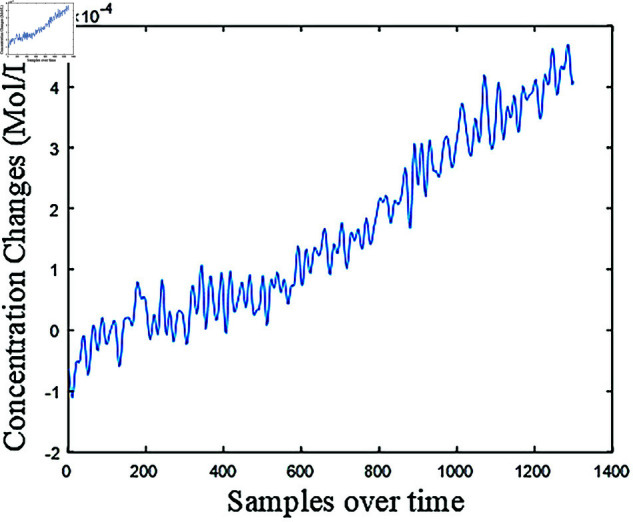
Interpolated fNIRS signal.

Each strategy was conducted separately for DeoxyHb, OxyHb, and Total-hemoglobin (TotalHb) trials. Linear interpolation, a type of interpolation used in biomedical signals [20], was first applied to the trials. This interpolation was used to standardize all signals to 1300 samples, which corresponds to the maximum number of samples observed within the trials. Fig 6 shows the interpolated fNIRS signal.

ICEEMDAN was applied following linear interpolation and represents an advanced version of the empirical mode decomposition (EMD) algorithm, which is a self-adaptive approach that utilizes an iterative approximation algorithm (elimination process) to analyze non-stationary and transient signals [21]. By decomposing signals into intrinsic mode functions (IMFs), it offers significant advantages over traditional time-frequency decomposition techniques. Unlike the Fourier, [22] and Wavelet Transforms [23–25], EMD provides adaptive decomposition without requiring assumptions of signal stationarity. This flexibility delivers superior time-frequency resolution while addressing common issues such as edge effects and mismatched basis functions. Furthermore, EMD extracts frequency-modulated signal components without the need for predefined window lengths, bandpass filter cutoffs, or the selection of a mother wavelet. Its local and adaptive nature makes it theoretically well-suited for capturing underlying physiological processes. However, earlier versions of the EMD faced limitations, including the generation of spurious modes, residual noise, and the final averaging problem. As a solution to these limitations, ICEEMDAN, a developed version of the EMD, introduces an enhanced decomposition framework that ensures greater robustness and reliability in signal processing. By leveraging its unique assets, we selected the ICEEMDAN as a suitable approach for analyzing non-stationary fNIRS signals. Given its strong noise minimization capability, ICEEMDAN effectively removes both natural and artificial noise, thereby enabling the extraction of IMFs that accurately reflect cognitive state-related features from complex neural activity signals [26]. In summary, ICEEMDAN enhances the neural interpretability of IMFs, reduces noise, and provides a more reliable signal decomposition framework for extracting meaningful cognitive state-related features [27].

The steps of the ICEEMDAN algorithm are provided below in Eqs 1–8:

1) The first mode estimation is given by:

*E*_1_(*x*) = *x*−*M*(*x*), for $x^{i}[n]=x[n]+\beta_{0}E_{1}\left(w^{\left(i\right)}\right)$, i=1, 2,..,L,

The first residue signal is calculated as:

$$r_{1}=mean(M(x^{i}[n]))$$ (1)

2) The first IMF is determined as:

$$IMF_{1}=x-r_{1}$$ (2)

3) The second residue is obtained by:

$$r_{2}=mean(M(r_{1}+\beta_{1}E_{2}(w^{\left(i\right)})))$$ (3)

and the second mode is computed as:

$$IMF_{2}=r_{1}-r_{2}$$ (4)

4) The remaining residual signals and IMFs are calculated iteratively as follows:

$$r_{j}=mean(M(r_{j-1}+\beta_{j-1}E_{j}(w^{\left(i\right)})))$$ (5)

and

$${IMF}_{j}=r_{j-1}-r_{j}$$ (6)

5) Repeat step 4 until all IMFs are obtained.

In the formulas above, x[n] represents the fNIRS signal, and M(.) denotes the operator that calculates the local average of the signal. *w*^*i*^ is white Gaussian noise with zero mean and unit variance, L is the number of realizations, and *E*_*k*_(.) is the *k*^*th*^ mode production operator derived via EMD. A constant $\beta_{j-1}$ is used to adjust the signal-to-noise ratio (SNR) between the residual and the added noise.

For the first iteration (j=1), $\beta_{0}$ defined as:

$$\beta_{0}=\epsilon_{0}std(x)/std(E_{1}(w^{\left(i\right)})$$ (7)

where std(.) represents the standard deviation operation and $\epsilon_{0}$ is the desired SNR between the input signal x[n] and the first added noise.

For $j>=2,\beta_{j}$ is given by,

$$\beta_{j}=\epsilon_{0}std(r_{k})$$ (8)

In this study, the ICEEMDAN parameters are the same as those used by Colominas *et al*. [28] for biomedical signals ($\epsilon_{0}=0.2$, L=100).

**Fig 7:**
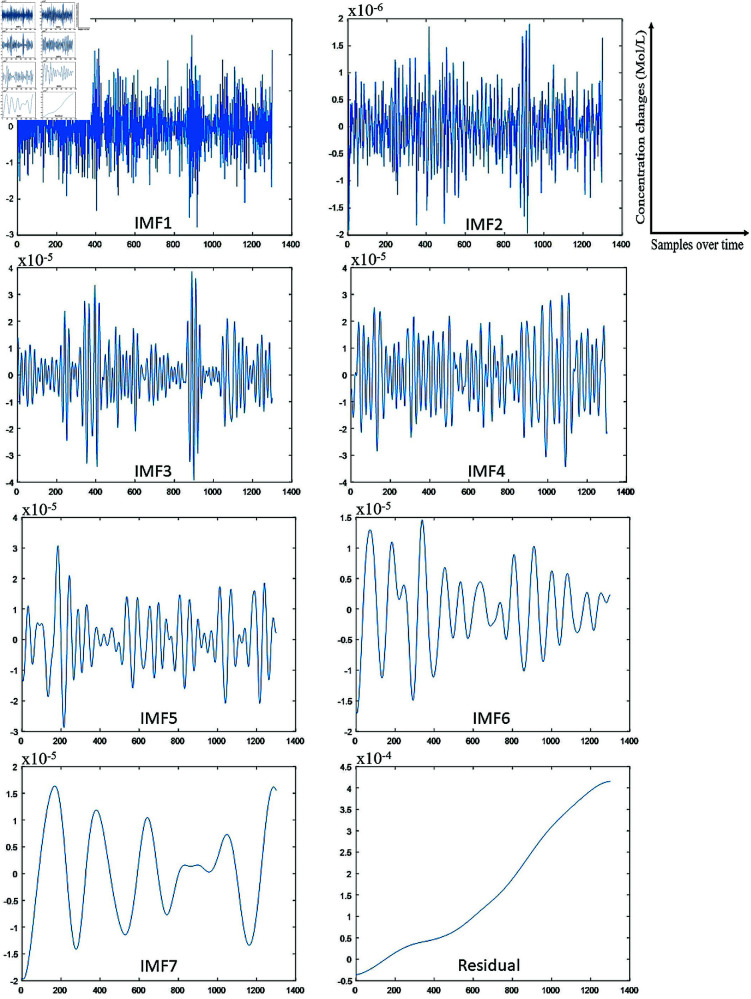
Decomposition of the fNIRS signal using ICEEMDAN.

The IMFs and residual obtained by applying the ICEEMDAN algorithm to the fNIRS signal are shown in Fig 7. In both figures, the x-axis represents the fNIRS samples over time, and the y-axis represents the concentration changes. The IMFs of all channels were obtained in the signal pre-processing stage.

#### Feature extraction

Feature extraction is the process of extracting the distinctive features of brain functions belonging to different classes and obtaining the feature vector. At this stage, the SAX coefficients were first calculated, followed by the application of a triple window shifting operation. SAX is a symbolic representation technique for time series that enables indexing with a lower-bound distance measure [29]. It transforms the original time series into a time-invariant representation, improving recognition accuracy and ensuring stability in neural activity analysis over time. Additionally, it provides a compact and interpretable representation, reducing computational complexity while preserving essential patterns in the data [30,31]. These advantages are particularly useful in the analysis of high-dimensional and noisy neurophysiological data, such as brain signals, where extracting stable and discriminative features is crucial for accurate classification. We selected SAX in our study due to its ability to efficiently encode time-series patterns while maintaining robustness against variations in signal morphology and above-aforementioned strong advantages.

This method converts a time series y[n] of length *n* into a symbolic sequence *y’* of sample length *w*. The SAX algorithm proceeds in three steps [32]:

1) The time series is normalized to have a mean of zero and a std of one using Eq 9.

$$y_{\text{new }}=\frac{y-\mu }{\sigma }$$ (9)

where μ is the mean and σ is the std of all samples in the time series y[n]. *y*_*new*_ represents the normalized sample values.

2) The normalized signal is divided into parts of equal size and a specified number of parts. The average value of each part is calculated. Each part is represented by its average value. This process is called piecewise aggregate approximation (PAA) [32].

3) Following the PAA process, symbol discretization is performed to create a symbolic sequence. Since the normalized time series will have a Gaussian distribution, it is easy to perform equal probability symbol discretization and determine the breakpoints [33]. These breakpoints can be determined from a statistical table [34].

**Fig 8:**
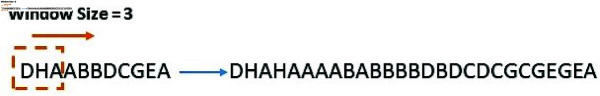
Triple window shifting operation.

The SAX algorithm segmented the trials into 100 segments of 13 samples, 650 segments of two samples, and 1300 segments of a single sample. Each segmentation was tested individually during the classification process to determine the optimal number of segments that yielded the highest CA. The number of symbols in SAX was selected to range from 5 to 8, inspired by the successful results reported by Lin *et al*. [33]. After the SAX process, the symbolic trials were augmented using a triple window shifting operation and then converted into numerical values from 1 to 8 (A=1, B=2, ..., H=8). Thus, the trials were prepared for statistical feature extraction. The shifting process is illustrated in Fig 8.

The most effective combination of statistical features was determined using sequential forward feature selection [35] among mean, variance, skewness, kurtosis, and std. The formulas for these statistical features are presented in Eqs 10–14, respectively.

$$Mean=\frac{\sum_{i=1}^{n}Z_{1}+Z_{2}+\ldots +Z_{n}}{n}$$ (10)

$$Variance=\frac{1}{n}\sum_{i=1}^{n}{\left(Z_{i}-\overset{―}{Z}\right)}^{2}$$ (11)

$$Skewness=\frac{\frac{1}{n}\sum_{i=1}^{n}{\left(Z_{i}-\overset{―}{x}\right)}^{3}}{(\sqrt{{\frac{1}{n}\sum_{i=1}^{n}{\left(Z_{i}-\overset{―}{Z}\right)}^{2})}^{3}}}$$ (12)

$$Kurtosis=\frac{\sum_{i=1}^{n}\frac{\left(Z_{i}-\overset{―}{Z}\right)}{n}}{{Std}^{4}}$$ (13)

$$Std=\sqrt{\frac{\sum {\left(Z_{i}-\overset{―}{Z}\right)}^{2}}{n}}$$ (14)

If we consider the output of the triple window shifting operation as z[n], $\overset{―}{z}$ is the mean of *z*, std is the standard deviation of *z*, and *n* is the number of data points.

The features extracted using the combination of skewness, kurtosis, and std, identified through forward feature selection, were utilized during the classification stage.

#### Classification

The extracted features were classified by the *k*-NN classifier. During the classifier training, the optimal *k* value was determined by searching within the range of 1 to 25. For each strategy, the total number of trials was divided into training and test sets, consisting of 70% and 30%, respectively. The trial distribution for Strategies 1 and 3 is presented in Table 1, while the distribution for Strategy 2 is provided in Table 2.

**Table 1:** Strategy 1 and Strategy 3 trial distribution.

		Understood Class		Not Understood Class	
Strategy	Total	Training	Test	Training	Test
1	450	174	74	141	61
3	274	122	52	70	30

**Table 2:** Strategy 2 trial distribution.

		Class 1		Class 2		Class 3	
Strategy	Total	Training	Test	Training	Test	Training	Test
2	450	92	40	70	30	153	65

Classifier performance was evaluated using the polygon area metric (PAM) algorithm, which evaluates classifier performance on irregular data [36]. Additionally, a total of 6195 combinations were created with single, double, triple, and four-channel combinations. Each of them was used during the classification stage. The computer processing time increased significantly when more than four-channel combinations were applied. Hence, the maximum number of channels in the combinations was limited to four. All training and test algorithms were performed in MATLAB R2022a environment on a 2.2 GHz Intel Core i7 processor-powered computer with 16GB, 2933 MHz DDR4 memory.

The *k*-NN is a popular, easy, and helpful algorithm for classifying binary and multiclass problems. In the classification stage, all the training trials are required to define the label of a test trial on the set of *A* labeled examples and predefined *B* classes. The *k*-NN algorithm measures the distances between the testing trial and all training trials to determine its nearest neighbors. *k*-NN uses a majority vote to define the class label of the testing trial set on the *k* nearest neighbor(s). Euclidean distance function calculates the metric distance between two data points. The distance of a test trial from a training trial can be calculated using the following equation:

$$D=\sqrt{{\left(X-X_{1}\right)}^{2}+{\left(Y-Y_{1}\right)}^{2}}$$ (15)

where *D* is distance, *X* and *Y* are the coordinates of a training trial, and *X*_1_ and *Y*_1_ are the coordinates of a testing trial.

CA, sensitivity (SE), specificity (SP), area under the curve (AUC), Jaccard index (J), and F-measure (FM) metrics were obtained by PAM. Thus, the classifier performance can be evaluated using PAM without needing various metrics.

For two-class problems, CA, SE, SP, J, FM, and precision can be calculated by finding the confusion matrix [37]. If one of these classes is named as positive and the other as negative, true positive (TP) refers to the number of correctly predicted positives, false positive (FP) to the number of negative samples incorrectly assigned to the positive class, true negative (TN) to the number of negative samples assigned to the correct class, and false negative (FN) to the number of positive samples assigned to the incorrect class. In this study, the positive class refers to the *understood* class, and the negative class refers to the *not understood* class. Eqs 16-21 show the formulas of the performance evaluation metrics.

$$CA=\frac{TP+TN}{TP+TN+FP+FN}$$ (16)

$$SE=\frac{TP}{TP+FN}$$ (17)

$$SP=\frac{TN}{TN+FP}$$ (18)

$$J=\frac{TP}{TP+FP+FN}$$ (19)

$$FM=\frac{2TP}{2TP+FP+FN}$$ (20)

$$Precision=\frac{TP}{TP+FP}$$ (21)

In addition to these metrics, the AUC metric is calculated as given in Eq 22.

$$AUC=\int_{0}^{1}f(x)dx$$ (22)

In this equation, f(x) represents a receiver operating characteristic curve, where the true positive rate is plotted as a function of the false positive rate for different cut-off points.

**Fig 9:**
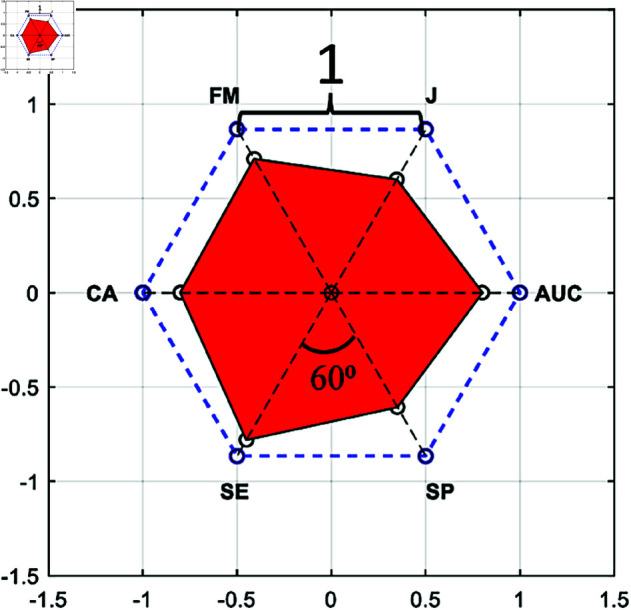
PAM graph.

PAM is calculated from the red area (RA) formed by the CA, SE, SP, J, FM, and AUC metrics falling on the lines drawn from the center to the edges of a regular hexagon with a side length of 1, as shown in Fig 9. RA consists of 6 triangles with an angle of 60 degrees. The area of each triangle is equal to half of the product of the length of two sides and the sinus of the angle between these sides (60 degrees). Hence, RA can be calculated as given in Eq 23. In this equation, *a*_*i*_ and *b*_*i*_ are the sides adjacent to the 60-degree angle of triangle i. The area of a regular hexagon with a side length of 1 is 2.59807, and since the PAM value is normalized between 0-1, the RA in the figure is divided by 2.59807, as given in Eq 24.

$$RA=\frac{\sqrt{3}}{4}\sum_{i=1}^{6}a_{i}b_{i}$$ (23)

$$PAM=\frac{RA}{2.59807}$$ (24)

## Results

**Fig 10:**
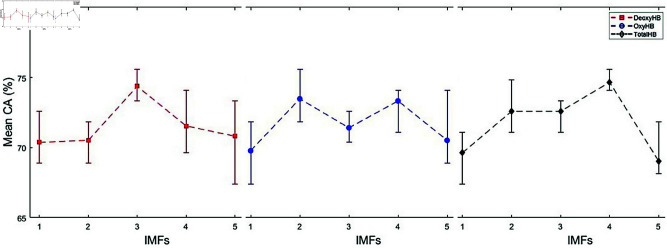
Comparison of mean CAs for DeoxyHb, OxyHb, and TotalHb trials across different IMFs in Strategy 1.

In this study, we proposed a method to determine the reading comprehension status of whole text in English using fNIRS signals. Due to the limited data and to demonstrate the robustness of the proposed method, the program was executed five times for each of the five different IMF values of DeoxyHb, OxyHb, and TotalHb trials using a randomized data selection procedure. We calculated the mean CAs by averaging the highest CAs obtained from five runs at each IMF value for each trial. The mean CAs for the three strategies are presented in Figs 10, 12, and 14. In these figures, the vertical lines represent the range of CAs, indicating the minimum and maximum CAs observed at each IMF value.

**Fig 11:**
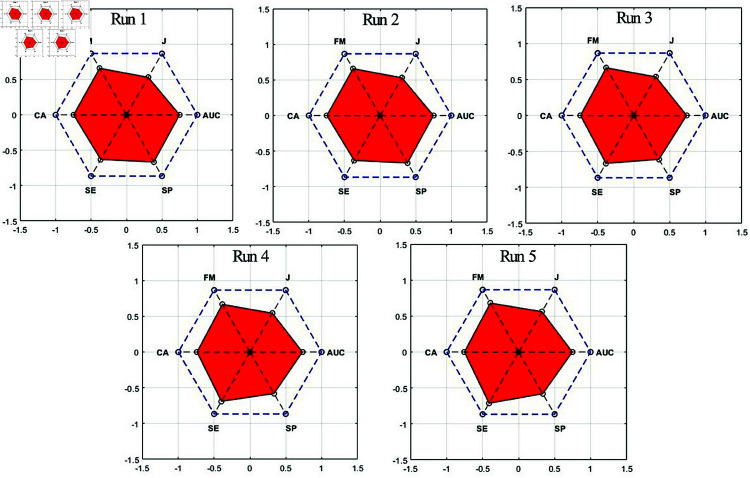
PAM graphs of the runs in Strategy 1.

**Fig 12:**
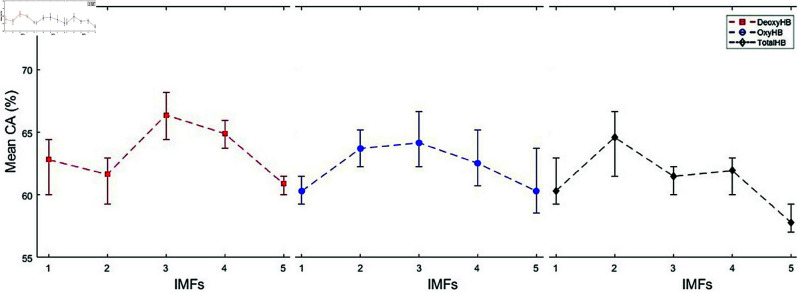
Comparison of mean CAs for DeoxyHb, OxyHb, and TotalHb trials across different IMFs in Strategy 2.

**Fig 13:**
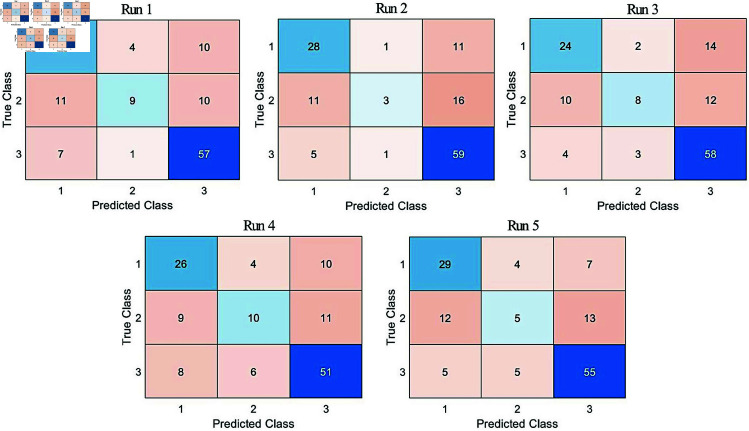
Confusion matrices of Strategy 2 for DeoxyHb $IMF_{3}$ trials across five runs.

**Fig 14:**
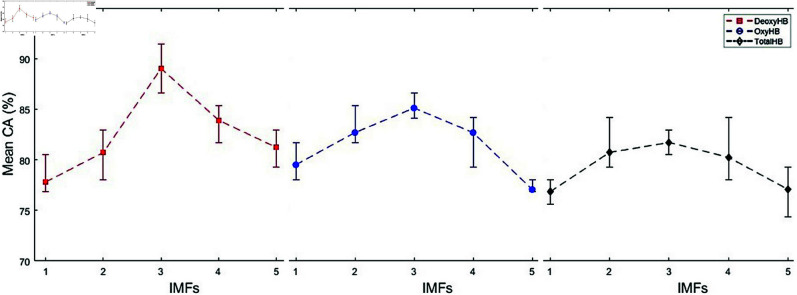
Comparison of mean CAs for DeoxyHb, OxyHb, and TotalHb trials across different IMFs in Strategy 3.

### Strategy 1: Trials labeled based on answers to multiple-choice questions

Strategy 1 presents the classification results for trials labeled based on responses to multiple-choice questions. The mean, minimum, and maximum CAs obtained are illustrated in Fig 10. The reported values were derived using 1300 segments and 8 symbols, as this combination yielded the highest CA. The highest mean CA achieved was 74.66% with a standard deviation value (SDV) of 0.62 for the TotalHb *IMF*_4_ trials. The best CA values over five runs ranged between 74.07% and 75.56%. Notably, in three of the five runs, the *understood* class exhibited a higher CA, whereas in the remaining two runs, the *not understood* class achieved a higher CA.

To assess the impact of segment numbers on CA, the number of segments was varied while keeping the number of symbols constant at 8. The results for TotalHb *IMF*_4_ trials are summarized in Table 3.

**Table 3:** Results of Strategy 1 for different numbers of segments in TotalHb $IMF_{4}$ trials.

Segment Number	Mean CA ± SDV (%)
100	72.74 ± 1.52
650	73.04 ± 2.00
1300	74.67 ± 0.62

To analyze the impact of different symbol numbers, the segment number was fixed at 1300, and symbol numbers were varied. The corresponding results are presented in Table 4.

**Table 4:** Results of Strategy 1 for different numbers of symbols in TotalHb $IMF_{4}$ trials.

Symbol Number	Mean CA ± SDV (%)
5	72.30 ± 1.93
6	72.59 ± 2.18
7	73.48 ± 2.28
8	74.67 ± 0.62

To provide a comprehensive evaluation of the model’s performance, we analyzed additional metrics, including PAM, SE, SP, AUC, J, and FM across five runs of TotalHb *IMF*_4_ trials, as illustrated in Table 5. The highest PAM value, 54.51%, was achieved in run 5. Table 6 presents the confusion matrices for TotalHb *IMF*_4_ trials across five runs, detailing TP, FN, TN, and FP values. All results presented in Tables 5 and 6 were obtained using 1300 segments and 8 symbols. The comparison of different metrics is effectively visualized in Fig 11. In addition to these metrics, we also calculated the precision values for each run as 79.41%, 79.41%, 76.00%, 74.65%, and 75.31%, respectively.

**Table 5:** PAM and the results of its constituent values for Strategy 1 on TotalHb $IMF_{4}$ trials across five runs.

Run	PAM (%)	CA (%)	SE (%)	SP (%)	AUC	J	FM
1	53.04	74.81	72.97	77.05	0.75	0.61	0.76
2	53.04	74.81	72.97	77.05	0.75	0.61	0.76
3	52.19	74.07	77.03	70.49	0.74	0.62	0.77
4	52.28	74.07	79.73	67.21	0.73	0.63	0.77
5	54.51	75.56	82.43	67.21	0.75	0.65	0.79

**Table 6:** Confusion matrices of Strategy 1 for TotalHb $IMF_{4}$ trials across five runs.

Run	TP	FN	TN	FP
1	54	20	47	14
2	54	20	47	14
3	57	17	43	18
4	59	15	41	20
5	61	13	41	20

### Strategy 2: Trials labeled based on SAS

Strategy 2 presents the classification results for trials labeled based on SAS. The mean, minimum, and maximum CAs obtained are illustrated in Fig 12. The highest CA was achieved using 650 segments and 8 symbols. The highest mean CA obtained for DeoxyHb *IMF*_3_ trials was 66.37% with a SDV of 1.35.

In the DeoxyHb *IMF*_3_ trials, mean CAs and SDVs were obtained by varying the number of segments while keeping the symbol number constant at 8. The results for DeoxyHb *IMF*_3_ trials are presented in Table 7. Table 8 shows the results obtained from 650 segments, using different numbers of symbols applied to the same trials.

**Table 7:** Results of Strategy 2 for different numbers of segments in DeoxyHb $IMF_{3}$ trials.

Segment Number	Mean CA ± SDV (%)
100	65.63 ± 2.90
650	66.37 ± 1.35
1300	65.93 ± 1.17

**Table 8:** Results of Strategy 2 for different numbers of symbol in DeoxyHb $IMF_{3}$ trials.

Symbol Number	Mean CA ± SDV (%)
5	63.70 ± 1.17
6	57.48 ± 1.24
7	63.56 ± 0.97
8	66.37 ± 1.35

The highest CAs for 650 segments and 8 symbols of DeoxyHb *IMF*_3_ trials across five runs ranged between 64.44% and 68.15%, and the results of all runs are presented in Table 9.

**Table 9:** Highest CA results of Strategy 2 for DeoxyHb $IMF_{3}$ trials across five runs.

Run	CA %
1	68.15
2	66.67
3	66.67
4	64.44
5	65.93

The confusion matrices corresponding to the highest CAs across the five runs of DeoxyHb *IMF*_3_ trials are presented in Fig 13. The confusion matrices in Strategy 2 indicate that the CAs for class *2* were significantly lower than those of the other classes. This class represents instances where participants demonstrated little understanding of the text. Based on these findings, the trials in Strategy 3 were refined by excluding instances where participants had little understanding of the text. This refinement aimed to obtain trials that more accurately represented the *understood* and *not understood* statuses, thereby enhancing classification performance.

### Strategy 3: Conditional *understood/not understood* trials

This strategy presents the classification results for trials labeled using double-validated labeling. The mean CAs for the *understood/not understood* trials are shown in Fig 14. All results were obtained using 1300 segments and 8 symbols, yielding the highest CA of 89.02% (SDV = ±1.92) for DeoxyHb *IMF*_3_ trials.

The DeoxyHb *IMF*_3_ trials mean CAs and SDVs were examined by varying the number of segments while applying 8 symbols. The results obtained with different numbers of segments are shown in Table 10.

**Table 10:** Results of Strategy 3 for different numbers of segments in DeoxyHb $IMF_{3}$ trials.

Segment Number	Mean CA ± SDV (%)
100	82.68 ± 2.0
650	85.85 ± 0.67
1300	89.02 ± 1.92

To analyze the impact of different symbol numbers, the segment number was fixed at 1300, and symbol numbers were varied. The corresponding results are presented in Table 11.

**Table 11:** Results of Strategy 3 for different numbers of symbols in DeoxyHb $IMF_{3}$ trials.

Symbol Number	Mean CA ± SDV (%)
5	86.58 ± 1.93
6	84.39 ± 2.18
7	85.36 ± 2.28
8	89.02 ± 1.92

The highest CAs across five runs, along with various metrics are detailed in Table 12. All results were achieved with 1300 segments and 8 symbols. The confusion matrices for the highest CAs across five runs for DeoxyHb *IMF*_3_ trials are presented in Table 13.

**Table 12:** PAM and the results of its constituent values for Strategy 3 on DeoxyHb $IMF_{3}$ trials across five runs.

Run	PAM (%)	CA (%)	SE (%)	SP (%)	AUC	J	FM
1	82.07	91.46	94.23	86.66	0.90	0.88	0.93
2	75.43	87.80	90.38	83.33	0.87	0.82	0.90
3	79.46	90.24	94.23	83.33	0.89	0.86	0.92
4	76.37	89.02	96.15	77.67	0.86	0.85	0.92
5	72.48	86.59	92.31	76.67	0.84	0.81	0.90

**Table 13:** Confusion matrices of Strategy 3 for DeoxyHb $IMF_{3}$ trials across five runs.

Run	TP	FN	TN	FP
1	49	3	26	4
2	47	5	25	5
3	49	3	25	5
4	50	2	23	7
5	48	4	23	7

**Fig 15:**
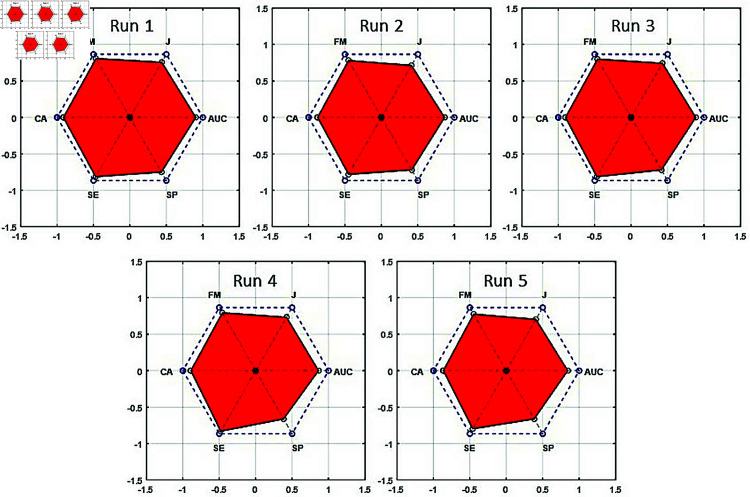
PAM graphs of the runs in Strategy 3.

Fig 15 presents the PAM graphs for the five runs with the highest CAs. As shown, the highest PAM rate of 82.07% was achieved in run 1. Generally, SP values were lower than SE, suggesting the model had a higher sensitivity in detecting the *understood* class compared to the *not understood* class. Based on the values presented in Table 13, the precision values for each run were calculated as 92.45%, 90.38%, 90.74%, 87.72%, and 87.27%, respectively.

In addition to the *k*-NN classifier, we compared the performance of the proposed method with other classifiers, including support vector machine (SVM), linear discriminant analysis (LDA), and decision tree (DT) algorithms. Table 14 summarizes the mean CAs of these classifiers across five runs on DeoxyHb IMF trials using Strategy 3. While the LDA classifier generally achieved consistent results with mean CAs between 72.19% and 76.59%, the DT classifier outperformed both SVM and LDA, achieving the highest CAs after the *k*-NN classifier, particularly for IMF3 and IMF4 trials. Notably, SVM yielded lower mean CAs compared to LDA, DT, and *k*-NN. Among all classifiers, the *k*-NN classifier achieved the highest CA of 89.02% for the IMF3 trials. The default settings of MATLAB were used for SVM, LDA, and DT classifiers.

**Table 14:** Mean CAs of SVM, LDA, DT, and *k*-NN classifiers for Strategy 3 on DeoxyHb IMFs across five runs.

IMF	LDA (%)	SVM (%)	DT (%)	*k*-NN (%)
1	72.68	68.05	74.88	77.81
2	73.35	67.56	76.59	80.73
3	76.59	64.87	82.20	89.02
4	72.19	68.29	82.44	83.91
5	75.37	71.71	75.37	81.22

## Conclusion and discussion

This study proposed an ICEEMDAN and SAX-based method for determining the reading comprehension status of the whole text in English and examined three different labeling strategies within this method. In this approach, ICEEMDAN provides the better identification of cognitive states associated with reading comprehension by effectively reducing both natural and artificial noise in non-stationary signals, owing to its strong noise minimization capability. To leverage the advantages of SAX, including robustness, stability, and low computational complexity, the time series of IMFs obtained through ICEEMDAN were converted into symbolic representations, enabling a more precise representation of cognitive processes.

As a result of strategy evaluations conducted using the proposed method, the optimal labeling strategy was determined. Our results indicate that CA was relatively lower in Strategy 1, where trials were labeled based on responses to multiple-choice questions, and in Strategy 2, where trials were labeled according to SAS. The lower CA in Strategy 2 may be attributed to subjective variability in participants’ SAS. Participants may either overestimate or underestimate their comprehension abilities, leading to less reliable ground truth labels. However, the implementation of double-validated labeling in Strategy 3 significantly improved CA, likely due to the enhanced labeling reliability achieved by integrating both objective task performance and subjective self-assessment. This finding suggests that relying solely on multiple-choice outcomes or self-reports may introduce biases or inaccuracies that compromise classification reliability. Using this refined labeling strategy and the proposed method, we calculated the mean value of PAM, CA, SE, SP, AUC, J, FM, and precision for DeoxyHb *IMF*_3_ trials as 77.16%, 89.02%, 93.20%, 81.40%, 0.87, 0.84, 0.91, and 89.71% respectively, using the *k*-NN classifier. These results are remarkable, with an SDV of 1.92. This low SDV indicates that the proposed method is stable and not significantly affected by random variation. These results align with previous neuroimaging studies emphasizing the role of the temporal lobe in reading comprehension processes, thus confirming the suitability of fNIRS for such cognitive assessments.

In addition to the *k*-NN classifier, alternative machine learning models, including SVM, LDA, and DT classifiers, were evaluated. This diversity in classifier evaluations strengthens the generalizability and robustness of the proposed feature extraction and classification framework.

Our findings highlighted that the proposed double-validated labeling strategy plays a crucial role in the development of an English reading comprehension training model. Furthermore, compared to previous studies that focused primarily on word- or sentence-level comprehension, our study advances the field by assessing reading comprehension at the whole-text level. This offers a richer and more relevant understanding of cognitive processing during naturalistic reading tasks under real-world conditions. In addition, we provide a unique dataset to facilitate future research in this domain.

In this study, analysis was limited to the temporal lobe channels recommended by previous literature for reading comprehension tasks. In future studies, the contribution of other brain lobes to the improvement of reading comprehension performance could be investigated.

Looking ahead, we aim to enhance CA performance through two key strategies: (1) increasing the dataset size and leveraging deep learning for more robust classification, and (2) exploring additional channel combinations with the aid of advanced computational resources. We believe that this method has strong potential to advance the field of reading comprehension assessment and contribute to future neurocognitive and educational research. The findings of this study lay the groundwork for future developments in brain-computer interface-based reading comprehension assessment systems, offering a novel approach that bridges neuroscience, cognitive science, and artificial intelligence.
